# Model of structural equations on the perception of aspects of school life and substance consumption as predictors of problem behavior in adolescents

**DOI:** 10.3389/fpsyt.2024.1386927

**Published:** 2024-03-26

**Authors:** Víctor J. Villanueva-Blasco, Bárbara González Amado, Ernesto Colomo Magaña, Sara Puig-Perez

**Affiliations:** ^1^ Faculty of Health Sciences, Valencian International University, Valencia, Spain; ^2^ Research Group on Health and Psycho-Social Adjustment (GI-SAPS), Valencian International University, Valencia, Spain; ^3^ Faculty of Science Education, University of Malaga, Malaga, Spain; ^4^ Research Group in Psychology and Quality of Life (PsiCal), Valencian International University, Valencia, Spain

**Keywords:** alcohol, tobacco, cannabis, problematic behaviors, school, sense of challenge, perception of oneself at school, value given to school

## Abstract

**Introduction:**

Problematic behavior and drug use are behaviors of social concern, especially in adolescence. There are school factors that can contribute to their development or prevent them. The objective was to establish explanatory models of the relationship between various school variables with the consumption of alcohol, tobacco and cannabis; determining its direct and/or indirect relationship with problematic behaviors.

**Materials and methods:**

The study was cross-sectional with a sample of 346 students (Main Age=14.81; 54% women) from secondary education. Structural Equation Model (SEM) were carried to analyze the relationship between the dimensions of the Scale of perception of aspects of school life and alcohol, tobacco and cannabis consume with the presence of Problematic Behavior.

**Results:**

Problem behavior was predicted by alcohol, tobacco and cannabis consume, and binge drinking. Different aspects of school life differentially predicted problem behavior and drug use. Sense of challenge was observed as having a reciprocal predictive relationship with problem behavior. Perception of oneself at school predicts both alcohol and tobacco use; while the value given to school predicts binge drinking.

**Discussion:**

These findings suggests that, when addressing problem behavior and drug use that affect minors in school and have repercussions on class dynamics and academic outcomes, we should not focus exclusively on these problem areas, but rather take a more integrated approach that includes modifying different school-related aspects that act as risk factors for these types of problems.

## Introduction

Antisocial behavior is one of the most common problems in adolescence (1). Although it tends to decrease with age (2), it can lead to antisocial behavior disorders if it persists into adulthood (3).

Several studies have highlighted the connection between antisocial behavior in minors and the use of alcohol, tobacco, and cannabis (4–8). Behaviors such as deliberately damaging public property and theft have been associated with risky substance use or using multiple drugs (6, 8). The meta-analysis conducted by Bennet et al. (4) concluded that users of certain drugs were 2.8 to 3.8 times more likely to engage in criminal behavior compared to non-users, and marijuana users were 1.5 times more likely to engage in criminal behavior. Rocca et al. (9) showed a clear link between cannabis use in the past month and criminal behavior in minors aged 12 to 16, suggesting a higher tendency for diverse criminal activities compared to alcohol consumers and a 2.6 times higher likelihood of committing some form of aggression.

Scientific evidence has shown that school and family are the most influential agents of primary socialization. School can either protect or pose a risk to the development of problem behaviors (10, 11) and the use of alcohol, tobacco, and cannabis (12, 13). Greater school commitment has been negatively related to substance use and being involved in fights (14). A good relationship between teachers and students, a student’s relationship with his or her classmates, opportunities for autonomy, and the clarity and consistency of rules are related to fewer behavioral problems (15, 16), given that they increase school satisfaction (17). In this regard, a positive school environment serves as a protective factor against the onset of substance use (18). Likewise, attachment or a sense of belonging to school (*school attachment*), a positive connection to it, and greater academic performance constitute protective factors against tobacco (19, 20) and cannabis consumption in adolescents (13, 19).

However, adolescence is a period in which academic motivation and achievement usually decrease (11). This lack of motivation, along with the punishments imposed by the school such as expulsions or suspensions, permanently affects the adolescent’s sense of identity and their belief in their academic abilities (11). This favors a progressive disengagement from school and can lead to them dropping out. In this sense, low academic achievement and early school failure increase the likelihood of the onset of substance use (21) and exhibiting problematic or criminal behaviors in early adulthood (22). Likewise, these school problems are associated with issues such as loss of motivation (23), high student-to-teacher ratio (24), school and classroom environment (25), grade repetition policies (26), disciplinary measures (27), or negative peer influence and bullying experienced at school (19, 28).

Decades ago, Wight (29) proposed the need for a change from traditional teaching methods that do not foster student involvement and from the idea that not meeting competencies required by the educational system labels a student as incapable. Wight (29) argued that the existing reality had enormous repercussions on motivation, given that the teaching model laid the entire load of guilt on the students themselves. The importance of careful attention to aspects of school life has been made evident in different studies, where analyses have linked a sense of achievement to motivation (27), school experiences (30), and student engagement with their studies (31) and where parental expectations and opinions about the role of the school are a proven factor in influencing the value that the student gives to schooling (32).

Consequently, the objective of the present study was to establish models that explain the relationship between different school variables (perception of oneself at school, the value given to school, and the sense of academic challenge) and the consumption of alcohol, tobacco, and cannabis, as well as their direct and/or indirect connection to problem behaviors. In this regard, two different explanatory models were proposed: one for alcohol consumption and binge drinking (as a pattern of abusive alcohol consumption) and another for the consumption of tobacco and cannabis. This decision was based on the high rate of alcohol consumption among adolescents compared to the lower rates of tobacco and cannabis consumption, as shown by epidemiological studies (33). In addition, there is a significant relationship between the consumption of tobacco and cannabis. Findings indicate that nine out of 10 individuals who have consumed cannabis in the last month have also consumed tobacco during this period, and four of 10 daily tobacco users have also consumed cannabis in the past 30 days (33).

## Method

### Study design

The study was cross-sectional with a sample of students from different schools and different grade levels of secondary education in Teruel (Spain). Four compulsory secondary education public schools were randomly selected (73.5% of the student population were enrolled in public schools). The directors of three of these four schools agreed to be part of the study.

### Participants

A total of 346 participants were recruited. The age span was 13 to 17 years (*M* = 14.81; *SD* = 0.68). Sex distribution was 46% (n = 159) boys and 54% (n = 187) girls.

### Instruments

#### Sociodemographic questionnaire

Developed *ad hoc*, it included data on the school, sex, and age.

#### Scale of perception of aspects of school life

The general scale (34) is composed of 25 items and three subscales that assess the following: a) perception of oneself at school or PO (items *a*–*i*, e.g., “*Sometimes I am not sure about what is expected of me at school*”), b) sense of challenge or SC (items *j*–*r*, e.g., “*I get a lot of satisfaction from solving the hard problems in class*”), and c) value given to school or VG (items *s*–*y*, e.g., “*A lot of what I do at school has nothing to do with real life*”). These subscales were measured on a 5-Likert-type scale according to the level of agreement with the statements given, where 0 means totally agreed and 4 totally disagree. Cronbach’s alpha in this sample was 0.73 for the PO subscale, 0.84 for the SC subscale, and 0.75 for the VG subscale.

#### Scale of problem behavior 

The instructions of the scale (34) provide a total score of problem behavior (PB) by requesting participants to indicate the answer that best matches how often, over the past 12 months, they have done each action expressed in the statements. The variable is measured on a Likert-type scale with five choices according to the level of agreement with the statements given. Scores range from 8 to 40 points, where a higher score indicates greater problem behavior. Cronbach’s alpha was 0.86 with the sample of this study.

#### Questionnaire on frequency of drug use

This was an *ad hoc* questionnaire that incorporated different scales to assess the frequency of alcohol consumption and binge drinking behavior over a 30-day time period. Alcohol consumption was registered using a 7-point Likert scale, as follows: 1 day (1), 2 days (2), 3 days (3), from 4 to 5 days (4), from 6 to 9 days (5), from 10 to 19 days (6), and 20 days or more (7). Regarding binge drinking behavior (BD), the 7-point Likert scale was used, as follows: none (0), one (1), two (2), three (3), four (4), five (5), and more than five times (6). Tobacco consumption (TC) was registered using a 4-point Likert scale, as follows: never (0), less than once a week (1), once a week, but not daily (2), and daily (3). Cannabis consumption (CC) was registered using a 6-point Likert scale, as follows: no day (0), 1 or 2 days (1), 3 to 5 days (2), 6 to 9 days (3), 10 to 19 days (4), and 20 or more days (5).

### Procedure

The data were collected during the 2019–2020 school year, between November and December 2019. Prior to administering the questionnaires, the schools were provided with an informational letter to be used for requesting informed consent from students’ legal guardians. The letter explained the voluntary nature of participation and that data were kept confidential through the use of an alphanumeric code. In the beginning, the students’ teacher was asked to confirm the guardians’ authorizations. Next, the study was presented to the students, briefly explaining the research and requesting their collaboration. The protocol for administering the battery of instruments was a 30- to 40-minute duration, carried out in the regular classroom during normal class hours, under supervision by the researcher.

Participation in this study was subject to the ethical standards of the Declaration of Helsinki (35) and the Spanish Organic Law 3/2018, dated December 5, Protection of Personal Data and the Guarantee of Digital Rights (36). The data were treated confidentially, respecting participants’ privacy. The study was approved by the Ethics Committee of the University of Santiago de Compostela (Spain) and the Ethics Committee of Clinical Research of Aragon (Spain).

### Statistical analysis

The data were introduced into SPSS version 26.0, from which reliability analyses were carried out for all the scales and subscales, as well as partial correlations in order to explore statistical models of the possible relationships between variables explored.

Structural equation modeling (SEM) was carried out through AMOS v.26 to analyze the relationship between the dimensions of the *scale of perception of aspects of school life* (VG, SC, and PO) and consumption of alcohol, tobacco, and cannabis with the presence of problem behavior. Structural equation model sensitivity was tested following Harring et al. (37) adding sex (boys *vs*. girls), age, and interculturality (ethnical minority yes *vs*. no) variables due to previous studies highlighting their relevance on alcohol, tobacco, and/or cannabis consumption (8, 38). To evaluate the fit of the SEM, the following were checked: the goodness-of-fit indices of χ^2^/df value, the comparative fit index (CFI), the goodness-of-fit index (GFI), the incremental fit index (IFI), the non-normalized fit index [Tucker–Lewis index (TLI)], the normalized fit index (NFI), and the root mean square error of approximation (RMSEA). Although the rest of the indices were examined, they are not presented in this study. It was set that a value lower than 3 shows a good fit of χ^2^/df value. Moreover, the cut-off point for the other indices examined was >0.95 for the CFI, GFI, IFI, and TLI to show an optimal fit; greater than 0.90 for the NFI value (39); and less than 0.06 for the RMSEA (39).

All *p*-values reported are two-tailed, and the level of significance was marked at *p* < 0.05. When not otherwise specified, results shown are means ± standard error of means.

## Results

### Correlation analyses

Results from the correlation analyses (**Table 1**) showed that the subscales of perception of aspects of school life (VG, SC, and PO) were correlated positively with each other. PB correlated positively with AC and BD and negatively with SC (all *p* < 0.01). PO and SC were negatively correlated with PB, TC, and CC within the last 30 days. Likewise, TC was positively correlated with CC in the last 30 days and with PB (all *p* < 0.05). Finally, the VG subscale of perception of aspects of school life was negatively correlated with PB (*p* < 0.01) and TC (*p* < 0.05) within the last 30 days, but not with CC.

**Table 1:** Descriptive data and correlation analyses between perception of aspects of school life; alcohol, tobacco, and cannabis consumption; binge drinking; and problem behaviors.

	*M*	*SD*	Perception of oneself	Sense of challenge	Value given to school	Problem behavior
Perception of oneself	34.09	4.70	–	–	–	–
Sense of challenge	31.80	6.18	0.42**	–	–	–
Value given to school	24.38	4.18	0.48**	0.68**	–	–
Problem behavior	13.88	5.63	−0.21**	−0.31**	−0.18**	–
Alcohol consumption	5.57	4.04	−0.00	−0.01	−0.01	0.18**
Binge drinking	2.22	4.62	−0.03	0.05	0.09	0.21**
Tobacco consumption	0.61	1.11	−0.23**	−0.12*	−0.12*	0.47**
Cannabis consumption	0.24	0.82	−0.17**	−0.13*	−0.10	0.35**

M, mean; SD, standard deviation.

*p < 0.05.

**p < 0.01.

### Structural equation model with alcohol and binge drinking variables

**Figure 1:**
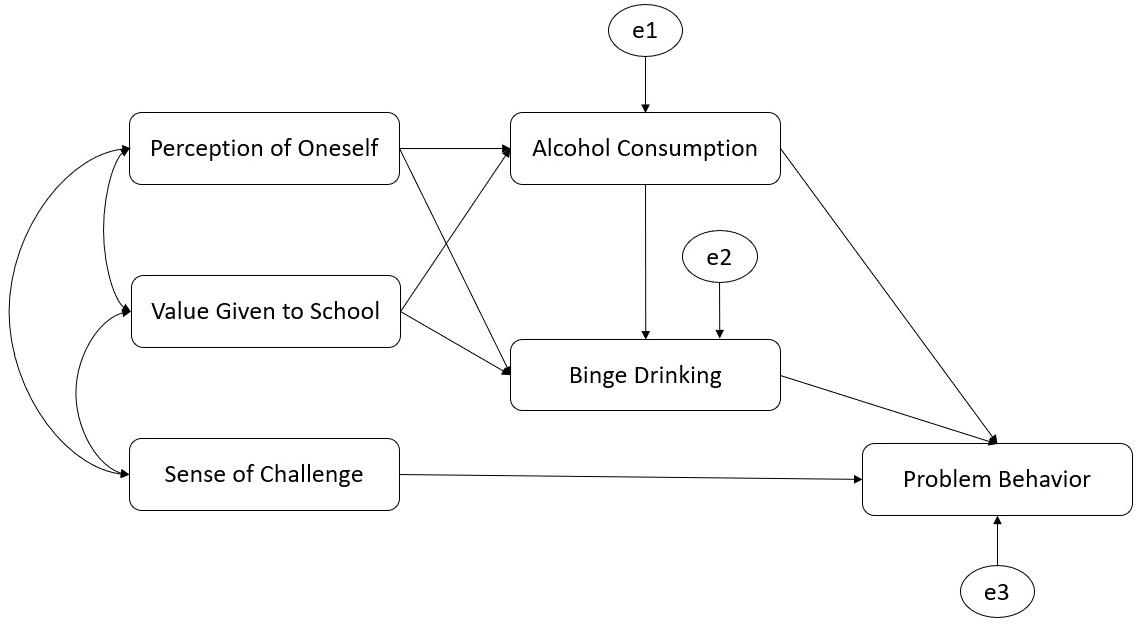
Structural equation model (SEM): aspects of school life, alcohol consumption, binge drinking, and problem behavior.

A SEM was tested on which the subscales of perception of aspects of school life (VG, SC, and PO) and alcohol behavior (AC and BD) predict PB. Considering the direct relationship observed between SC and PB on correlation analyses, the possible mediatory role of alcohol behavior (AC and BD) was explored only in the relationship of VG and PO with PB. Considering that the chi-squared test [χ^2^(4) = 3.54, *p* = 0.47] can be biased due to sample size or other factors, the corrected measure was used considering the degrees of freedom (χ^2^/df = 0.886), which showed a good model fit, as it was less than 3. In this line, the other fit indices confirmed an optimal fit model with a CFI value > 0.999, a GFI of 0.997, an IFI > 0.999, a TLI > 0.999, and an NFI of 0.994. Therefore, all of them showed values higher than the cut-off stated. Regarding the RMSEA, it showed a value < 0.01, which reflects a good model fit. The model tested was explored based on correlation analyses performed previously and is shown in **Figure 1**. No significant changes were observed in the model estimates when sex, age, and interculturality were introduced as control variables. Therefore, we can infer that there are no confounding effects. The covariance estimated between the three subscales of the Scale of perception of aspects of school life (VG, SC and PO) showed a positive and significant correlation (see **Table 2**).

**Table 2:** Covariance weights of the hypothesized relationships.

Relationships between covariances			Covariance weight			
			Estimate	SE	CR	*p*
Sense of challenge	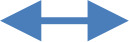	Perception of oneself	9.480	1.171	8.099	***
Perception of oneself	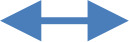	Value given to school	17.570	1.677	10.476	***
Sense of challenge	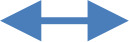	Value given to school	12.290	1.692	7.262	***

SE, standard error; CR, critical ratio.

***p < 0.001.

**Figure 2:**
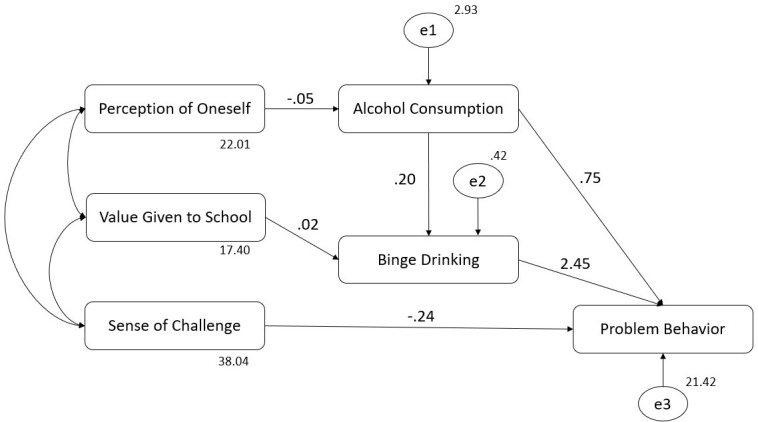
Structural equation model (SEM) with estimated parameters: aspects of school life, alcohol consumption, binge drinking, and problem behavior.

**Table 3** shows the weight of the hypothesized regression in detail. All significant relationships shown in the table are those indicated in **Figure 2**, meaning that only SC showed a negative direct relationship with PB. There was no significant direct relationship of VG and PO with PB. Notwithstanding, SC, VG, and PO showed a significant and negative join effect over PB. Regarding indirect relationships, PO was indirectly related to PB in two ways: i) being negatively related to AC, which in turn was positively related to PB, and ii) being negatively related to AC, which was positively related to BD, which in turn was positively related to PB. In contrast, VG was related to PB only through its positive relationship to BD, which was positively related to PB. No significant relationships were found between VG and AC.

**Table 3:** Regression weights of the hypothesized relationships.

Relationships between variables			Regression weight			
			Estimate	SE	CR	*p*
PB	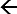	SC	−0.245	0.040	−6.044	***
AC	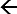	PO	−0.046	0.022	−2.028	*
AC	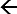	VG	−0.004	0.025	−0.161	0.872
BD	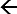	AC	0.203	0.020	9.957	***
BD	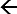	VG	0.019	0.010	1.987	*
BD	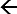	PO	−0.017	0.009	−1.984	*
PB	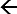	AC	0.755	0.165	4.580	***
PB	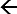	BD	2.450	0.381	6.423	***

PB, problem behavior; SC, sense of challenge; VG, value given to school; PO, perception of oneself at school; AC, alcohol consumption in the last 30 days; BD, binge drinking in the last 30 days; SE, standard error; CR, critical ratio.

*p < 0.05.

***p < 0.001.

Meaning of arrow: direction of the relationship.

### Structural equation model with tobacco and cannabis variables

**Figure 3:**
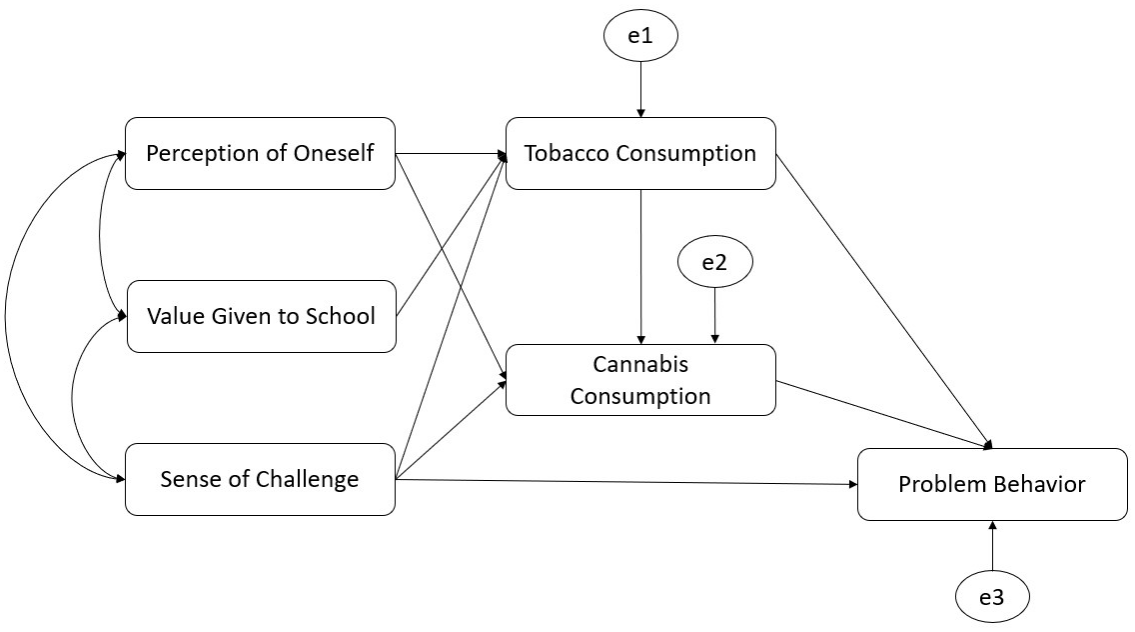
Structural equation model (SEM): aspects of school life, tobacco and cannabis consumption, and problem behavior.

The SEM tested the direct and indirect relationships of the *scale of perception of aspects of school life* (VG, SC, and PO) to PB through TC and CC through three paths: i) direct relationship of VG, SC, and PO with PB; ii) indirect relationship of VG, SC, and PO with PB through TC; and iii) indirect relationship of PB through TC and CC. Considering that the chi-squared test [χ^2^(3) = 2.66, *p* = 0.45] can be biased due to sample size or other, the corrected measure was used taking into account the degrees of freedom (χ^2^/df = 0.888), which showed a good model fit, as it was less than 3. In this line, the other fit indices confirmed an optimal fit model with a CFI value >0.999, a GFI of 0.997, an IFI >0.999, a TLI >0.999, and an NFI of 0.995. Therefore, all of them showed values higher than the cut-off stated. Regarding the RMSEA, it showed a value of <0.001, which reflects a good model fit. The model tested was explored based on correlation analyses performed previously and is shown in **Figure 3**. No significant changes were observed in the model estimates when sex, age, and interculturality were introduced as control variables. Therefore, we can infer that there are no confounding effects.

**Figure 4:**
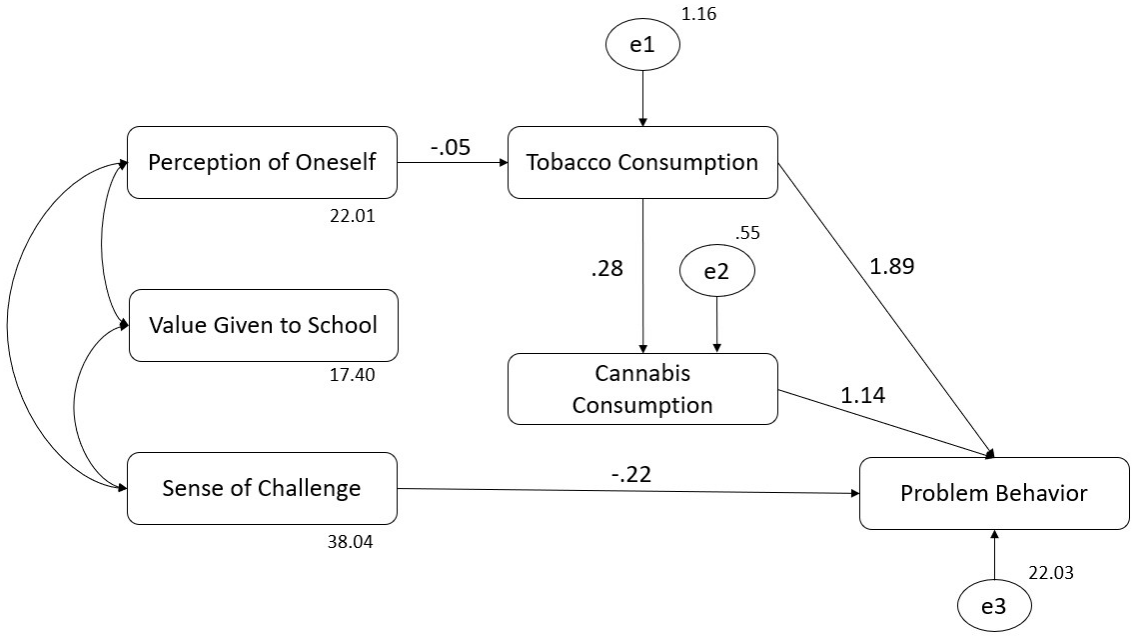
Structural equation model (SEM) with estimated parameters: aspects of school life, tobacco and cannabis consumption, and problem behavior.

**Figure 4** shows the model tested with values of the estimated parameters in the model after its re-specification. The correlation parameters, covariance, regression weights, and percentage of variance were collected. Only significant relationships were marked in the figure, with all of them significantly different from zero.

**Table 4** shows covariance estimated between the three subscales of the *scale of perception of aspects of school life* (VG, SC, and PO). All correlations showed were significant.

**Table 4:** Covariance weights of the hypothesized relationships.

Relationships between covariances			Covariance weight			
			Estimate	SE	CR	*p*
Sense of challenge	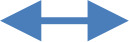	Perception of oneself	12.290	1.692	7.262	***
Perception of oneself	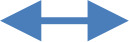	Value given to school	9.480	1.171	8.099	***
Sense of challenge	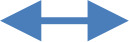	Value given to school	17.570	1.677	10.476	***

SE, standard error; CR, critical ratio.

***p < 0.001.

**Table 5** shows the weight of the hypothesized regression in detail. All the relationships shown in **Table 5** are those indicated in **Figure 4**, and all of them were significant, meaning that only SC showed a negative direct relationship with PB. There was no significant direct relationship of VG and PO with PB. However, VG, PO, and SC had a significant and negative join effect over PB. Regarding indirect relationships, PO was indirectly related to PB in two ways: i) being negatively related to TC, which in turn was positively related to PB, and ii) being negatively related to TC, which was positively related to CC, which in turn was positively related to PB. In contrast, there were no significant indirect relationships of VG and SC with PB through TC and/or CC.

**Table 5:** Regression weights of the hypothesized relationships.

Relationships between variables			Regression weight			
			Estimate	SE	CR	*p*
Tobacco consumption	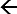	Perception of oneself	−0.051	0.014	−3.577	***
Tobacco consumption	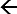	Value given to school	0.002	0.020	0.101	0.920
Tobacco consumption	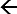	Sense of challenge	−0.007	0.013	−0.530	0.596
Cannabis consumption	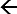	Tobacco consumption	0.278	0.037	7.493	***
Cannabis consumption	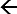	Perception of oneself	−0.011	0.010	−1.124	0.261
Cannabis consumption	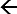	Sense of challenge	−0.008	0.007	−1.126	0.260
Problem behavior	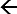	Tobacco consumption	1.887	0.250	7.556	***
Problem behavior	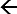	Cannabis consumption	1.138	0.339	3.357	***
Problem behavior	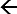	Sense of challenge	−0.218	0.041	−5.257	***

SE, standard error; CR, critical ratio.

***p < 0.001.

Meaning of arrow: direction of the relationship.

## Discussion

The objective of the present study was to establish models that explain the relationship between different school variables (perception of oneself at school, the value given to school, and the sense of academic challenge) and the consumption of alcohol, tobacco, and cannabis, as well as their direct and/or indirect connection to problem behaviors. The findings confirm the relevance of various aspects of school life that predict both drug use and problem behavior. This finding clearly highlights the need to complement student-focused interventions with structural interventions related to the prevailing educational model as well as with school environmental characteristics, such as school policy.

In consonance with previous literature, problem behavior was predicted by alcohol, tobacco, and cannabis consumption and binge drinking (3, 5, 40, 41). However, different aspects of school life differentially predicted problem behavior and drug use. Our results thus concur with findings from other studies, where sense of challenge was observed as having a reciprocal predictive relationship with problem behavior (17, 42, 43). For its part, self-perception at school predicts both alcohol and tobacco consumption, while the value given to school predicts binge drinking. These findings are new compared to the existing literature. It would be interesting to further study and confirm this relationship, as well as explore its explanation.

These findings suggest that, when addressing problem behavior and drug use that affect minors in school and have repercussions on class dynamics and academic outcomes, we should not focus exclusively on these problem areas but rather take a more integrated approach that includes modifying different school-related aspects that act as risk factors for these types of problems.

If perception of school-related aspects is a factor that favors or discourages the presence of antisocial behaviors and drug use, then the educational act cannot focus only on (cognitive) academic achievement and outcomes. This would be a partial view of educational reality; in the case of school failure, it contributes to a loss of expectations and rejection toward school (44), encouraging the appearance of antisocial behaviors in the educational context. Instead, one should work on students’ identity construction (45) through the implementation of attitudes that create a positive predisposition toward education and give priority to students’ perceived self-efficacy (46) and interests (47). However, as Wang and Eccles (48) maintain, schools are not always able to satisfy the psychological needs of adolescents.

Concerning how to foster student engagement in school, Sciarra and Seirup (49) indicated that this is strongly related to context variables, such as a) the size of the school, where small is preferable; b) class size, where a smaller number of students per classroom is beneficial; c) the type of teaching methodology and relational dynamics in the classroom, with preference to cooperative dynamics that emphasize shared responsibility, common goals, and decisions by consensus; d) power relations in the classroom, where there is clarity and flexibility of rules, and student–teacher roles are oriented toward cooperation and teacher support; and e) other aspects like acceptance of classmates, and assignments and learning that relate to real life.

The job of the school, then, is not limited exclusively to propaedeutic purposes pertaining to academic achievement and the acquisition of practical capacities of a vocational sort (50), but it involves learning that prepares one for life and makes life meaningful (51–53). If school is to provide training for life, it cannot be detached from it. Schools face the challenge of consolidating educational designs that are founded on a community-wide morality (54). In this way, our aim is to incorporate educational theories that encourage students’ involvement in the formative process from a whole-person perspective (considering social, affective, and cognitive aspects), including theories of participatory education (29), education for social change (55), personalized education (56), and education adapted to the 21st century (57). For learners to have a share in their own progress and achievement has a positive impact on the self-image they acquire in the educational sphere, in their motivation toward the teaching–learning process, and in their perception of school. This fact encourages a positive evaluation of the school context, benefitting social reality in two realms: the academic, where society becomes equipped with greater competencies, tools, and skills for dealing with the different problems and difficulties that life presents; and the ethical–moral, reducing behaviors that negatively affect peaceful coexistence between persons and the values that they hold (freedom, tolerance, justice, respect, etc.).

In the face of substance use or problem behavior, educational institutions tend to implement measures aimed at modifying students’ behavior. These measures place the students themselves as the focus of the problem, as the sole responsible parties for their conduct, thus fostering the development of stigma as problematic students. However, educational institutions have a significant responsibility in deciding which preventive programs to incorporate into their policy. A recent systematic review of school-based preventive programs in Spain (58) indicates that only 37.5% are evaluated. This translates into an inadequate response or unknown effect regarding a health issue, such as substance use, which also predicts problem behavior. Consequently, educational institutions should only incorporate classroom interventions with proven efficacy in preventive programs. Likewise, antismoking school policies were considered a relevant factor in reducing the relationship between antisocial peers and tobacco consumption. It moderates that relationship through monitoring and sanctioning smoking behaviors while students are on campus and influencing their attitudes toward tobacco consumption (20).

In conclusion, interventions in the school context must focus not only on the subjective or personal characteristics of students but also on school environment factors.

Regarding study limitations, on the one hand, there are limitations relating to the sample, its size, and geographic origin. It would be interesting to obtain results from other geographic regions, as well as work with a larger sample. On the other hand, while we have realized a cross-sectional study, it would be useful to carry out longitudinal studies that provide an understanding of the relational dynamics of these variables throughout the entire developmental period of adolescence. Critical approaches sustain that research on learning and school cannot overlook questions such as school values and activities, even though this creates enormous difficulties for analysis. For this reason, we consider it equally important to include these variables in future studies.

## Data availability statement

The raw data supporting the conclusions of this article will be made available by the authors, without undue reservation.

## Ethics statement

The studies involving humans were approved by Committee of Ethics of the University of Santiago de Compostela (Spain) and the Ethics Committee of Clinical Research of Aragon (CEICA) (Spain). The studies were conducted in accordance with the local legislation and institutional requirements. Written informed consent for participation in this study was provided by the participants’ legal guardians/next of kin.

## Author contributions

VV: Conceptualization, Data curation, Investigation, Writing – original draft, Writing – review & editing, Funding acquisition, Methodology, Project administration, Resources, Software, Supervision, Validation, Visualization. BG: Visualization, Writing – original draft, Writing – review & editing. EC: Writing – original draft, Writing – review & editing, Conceptualization. SP: Writing – original draft, Writing – review & editing, Data curation, Formal analysis, Software, Supervision, Validation, Visualization.
